# The Novel Functions of the PLC/PKC/PKD Signaling Axis in G Protein-Coupled Receptor-Mediated Chemotaxis of Neutrophils

**DOI:** 10.1155/2015/817604

**Published:** 2015-10-29

**Authors:** Xuehua Xu, Tian Jin

**Affiliations:** Chemotaxis Signal Section, Laboratory of Immunogenetics, National Institute of Allergy and Infectious Diseases, National Institutes of Health, Rockville, MD 20852, USA

## Abstract

Chemotaxis, a directional cell migration guided by extracellular chemoattractant gradients, plays an essential role in the recruitment of neutrophils to sites of inflammation. Chemotaxis is mediated by the G protein-coupled receptor (GPCR) signaling pathway. Extracellular stimuli trigger activation of the PLC/PKC/PKD signaling axis, which controls several signaling pathways. Here, we concentrate on the novel functions of PLC/PKC/PKD signaling in GPCR-mediated chemotaxis of neutrophils.

## 1. **Introduction**

Neutrophils, also known as polymorphonuclear leukocytes (PMNs), are short-lived and highly specialized immune cells that form the first line of defense against bacterial and fungal infections [1]. Not only are neutrophils an essential part of the innate immune system, but activated neutrophils also secrete a number of cytokines and chemokines to help to shape lymphocyte-oriented adaptive immunity [2, 3]. The recruitment of neutrophils to inflammatory sites is through a cellular process known as chemotaxis, directional cell migration guided by extracellular chemoattractant gradients [4]. Rapid recruitment of neutrophils is crucial for host defense; however, excessive recruitment of neutrophils into healthy tissues causes damage and inflammatory diseases such as asthma and arthritis [5, 6]. Thus, neutrophil chemotaxis is tightly controlled* in vivo *through chemoattractants and their receptors.

**Figure 1:**
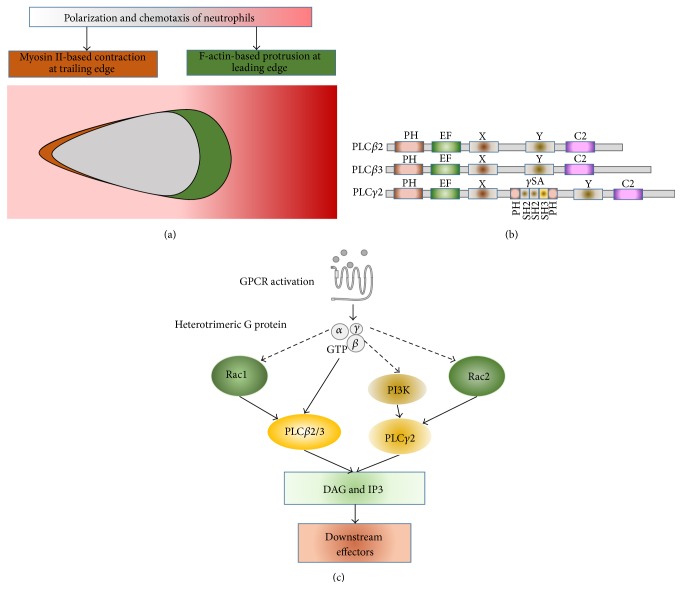
PLC isoforms and their signaling pathways in neutrophils. (a) F-actin-based protrusion in the leading edge and myosin-based contraction in the trailing edge of chemotaxing neutrophil cell. (b) Scheme shows the domain compositions of PLC isoforms expressed in neutrophils. Scheme shows the PH domain, EF-hand motifs, catalytic X and Y domains, and C2 domain in all PLC isoforms. In addition to the domains indicated above, PLC*γ*2 is characterized by the insertion of a highly structured region (PLC*γ*-specific array, *γ*SA), which is comprised of a split PH domain flanking two tandem SH2 domains and an SH3 domain between the two halves of a TIM-barrel catalytic domain. (c) Signaling pathways which activate PLC isoforms in neutrophils.

Chemotaxing neutrophils display a polarized morphology in a chemoattractant gradient (Figure 1(a)). They extend their leading edges by assembling a force-generating actin network beneath the plasma membrane [7, 8]. Actin also collaborates with myosin to retract the rear of migrating cells and to prevent errant pseudopod extension [7]. Neutrophils detect and move toward chemoattractant gradients by G protein-coupled receptor (GPCR) signaling pathways [4]. The most important GPCRs expressed in neutrophils include formyl-peptide receptors (FPR1/2/3), classical chemoattractant receptors (BLT1/2, PAFR, and C5aR), and chemokine receptors (CXCR1/2 and CCR1/2) [9]. The engagement of chemoattractants with their GPCRs triggers dissociation/activation of the GPCR-specific G*α* subunit from the G*βγ* dimer [10, 11]. Both G*α* and G*βγ* activate downstream effectors, such as phospholipase C (PLC) [12]. It has been shown that G*α*i and G*α*12/13 are involved in neutrophil chemotaxis [11, 13] although the coupling mechanism of GPCRs and their specific G*α*/*βγ* remains unclear. Over the last decade, multiple signaling pathways have been revealed to control GPCR-mediated organization of actin cytoskeleton in directional cell migration [8]. At the leading edge, signaling pathways control the activity of Arp2/3 complexes that initiate the formation of new branches of actin filaments. In neutrophils, GPCRs/G protein activation triggers multiple signaling pathways to activate the Rho family of small GTPases (cdc42 and Rac1/2) to promote the growth of actin filaments (F-actin) [12, 14–19]. GPCR activation also regulates the activity of cofilin, the F-actin depolymerization factor, to facilitate the rapid growth of F-actin in the leading edge [20–23]. Spatial-temporal activation of different signaling pathways for precisely controlled cell migration has just begun to be revealed.

PLC activation is an early event in response to numerous extracellular stimuli [24]. Upon activation, PLC produces two important second messengers: diacylglycerol (DAG) and inositol trisphosphate (IP_3_). Both DAG and IP_3_ play important roles in many signaling pathways, including the activation of protein kinase C (PKC) and protein kinase D (PKD) and the induction of calcium influx [12, 21, 23, 25]. It was shown that the signaling axis of PLC/PKC/PKD plays important roles in many signaling pathways [26]. In this review, we summarize the novel functions of the PLC/PKC/PKD signaling axis in GPCR-mediated chemotaxis of neutrophils.

## 2. **PLC Signaling Is Required for GPCR-Mediated Neutrophil Chemotaxis**

In response to various extracellular stimuli, PLC produces DAG and IP_3_, which mediate multiple downstream pathways. In mammals, there are 13 phosphatidylinositide-specific PLCs that are divided into 6 subgroups: PLC*β*, PLC*γ*, PLC*δ*, PLC*ε*, PLC*ξ*, and PLC*η* [24, 27–32]. Several excellent reviews have summarized the structures and activation mechanisms of PLC isoforms [24, 29, 33]. Mammalian neutrophils express PLC*β*2, PLC*β*3, PLC*γ*2, and PLC*ε* (Figure 1(b)). In murine neutrophils, chemoattractant stimulation robustly activates both PLC*β*2 and PLC*β*3 [12]. However, the evidence of PLC signaling function in neutrophil chemotaxis is contradictory. Early studies reported that murine neutrophils lacking both PLC*β*2 and PLC*β*3 are still able to chemotax [12]. Surprisingly, some leukocytes with a single PLC*β*2 deficiency actually have enhanced chemotaxis [34], indicating that PLC signaling might not play essential role in neutrophil chemotaxis. However, a PLC*β*/PI3K*γ*/GSK3 signaling pathway has been shown to regulate the activity of cofilin phosphatase slingshot 2 (SSH2) and control neutrophil polarization and chemotaxis [21]. The authors further investigated* in vitro* chemotaxis behavior of murine neutrophils with PLC*β*2 deficiency and suggested that the normal chemotaxis behavior of these murine neutrophils was rather context- and assay-dependent. A recent study has shown that when PLC activity is inhibited with the PLC inhibitor U73122, chemotaxis of human neutrophils is reduced, suggesting an essential role of PLC signaling in neutrophil chemotaxis [23]. Thus, PLC signaling appears to play a complicated role in neutrophil chemotaxis that is still not well understood.

In a chemoattractant gradient, PLC is recruited and activated at the leading edges of chemotaxing cells, suggesting its likely role in the remodeling of the actin cytoskeleton [23]. In neutrophils, GPCRs activate PLC*β* through several mechanisms (Figure 1(c)). First, chemoattractant stimulation may trigger PLC*β* activation through direct interaction with and activation by released heterotrimeric G proteins [12, 34]. Structural insights into GPCR-mediated PLC*β* activation have been summarized in a recent review [33]. Although there are currently no reported structures of a G*βγ*-PLC*β* complex that could shed light on the molecular basis for their interaction and activation, many studies have sought to map the interface of their interaction. GDP-bound G*α*i can inhibit PLC*β* activation, suggesting a common protein interaction interface on G*βγ* [35]. Beside direct activation by heterotrimeric G protein, PLC*β*2/3 might be activated by another mechanism. For example, in insect cells, PLC*β*2/3 is activated by small GTPase Rac1 [36]. The crystal structure of the Rac1-PLC*β*2 catalytic core complex has shown that the PH domain is the sole Rac1 binding site on PLC*β*2 and that the PH domain-mediated Rac1 interaction is sufficient to activate PLC*β*2/3 [37]. In neutrophils, chemoattractant stimuli trigger robust activation of Rac1 [38]. Moreover, in a chemoattractant gradient, the activated Rac1 localizes at the leading edge where PLC*β*2 is highly activated in chemotaxing neutrophils [39]. Rac1-mediated PLC*β* activation might provide an activation mechanism that is independent of GPCR or heterotrimeric G protein. It is intriguing to understand Rac1-mediated spatiotemporal activation of PLC*β* and its possible function in neutrophil chemotaxis.

PLC*γ*2 also plays critical roles in integrin- and Fc receptor-mediated neutrophil functions, such as respiratory burst, degranulation, and cell spreading* in vitro* [22]. PLC*γ*1 is ubiquitously expressed and is mainly activated downstream of growth factor stimulation, including stimulation by platelet derived growth factor (PDGF), vascular endothelial growth factor (VEGF), epidermal growth factor (EGF), and fibroblast growth factor (FGF). PLC*γ*2 is predominantly expressed in hematopoietic cells and is activated by immune cell receptors such as B cell and Fc receptors. PLC*γ*1 and PLC*γ*2 share similar domain composition and molecular structure. The activation mechanisms and functions of PLC*γ*1/2 have been well summarized [29]. Recently, it has been shown that chemokine GPCRs also mediate the membrane targeting and subsequent activation of PLC*γ*2 in a phosphoinositide 3-kinase- (PI3K-) dependent manner [23]. This result is consistent with the finding that PLC*γ*1 activation is a consequence of the binding between the PH domain of PLC*γ*1 and PIP_3_ produced on the membrane [40]. In addition, PLC*γ*2 might also be activated through another mechanism. It has been reported that PLC*γ*2 is specifically activated by Rac2 [41, 42]. In neutrophils, the engagement of chemoattractants with their GPCRs triggers the activation of Rac2 [18, 38]. The Rac1/2-mediated PLC*β*/*γ* activation adds another layer of complexity to the existing signaling networks of PLC signaling. GPCR-mediated PLC*γ*2 activation in neutrophils might provide an explanation for the normal chemotaxis behavior in murine neutrophils with single or double PLC*β*2/3 deficiency. However, the chemotaxis behavior of mammalian neutrophils lacking PLC*γ*2 remains unknown. Thus, it is difficult to evaluate the PLC isoform, and its activation is more important for neutrophil chemotaxis. Also, as a scaffold protein with numerous interacting partners, it is unlikely that GPCR- or Rac2-mediated PLC*γ*2 activation serves solely as the backup for PLC*β*2/3 in neutrophils. Further investigation is urgently needed to understand the role of PLC*γ*2 in neutrophil chemotaxis.

Neutrophils also express a low level of PLC*ε*, which is activated by GPCR and GPCR-regulated small GTPases, including Ras and Rap [24, 28, 43]. Recently, it was shown that PLC*ε* plays a crucial role in the neutrophil-associated inflammatory response [44]. In PLC*ε* ^−/−^ mice, neutrophil infiltration is remarkably suppressed. Future work is needed to elucidate the temporal and spatial activation profiles of each PLC isoform and their molecular mechanisms and subsequent effects on neutrophil chemotaxis.

## 3. **PKC Isoforms Play Different Roles in the Regulation of Neutrophil Chemotaxis**

**Figure 2:**
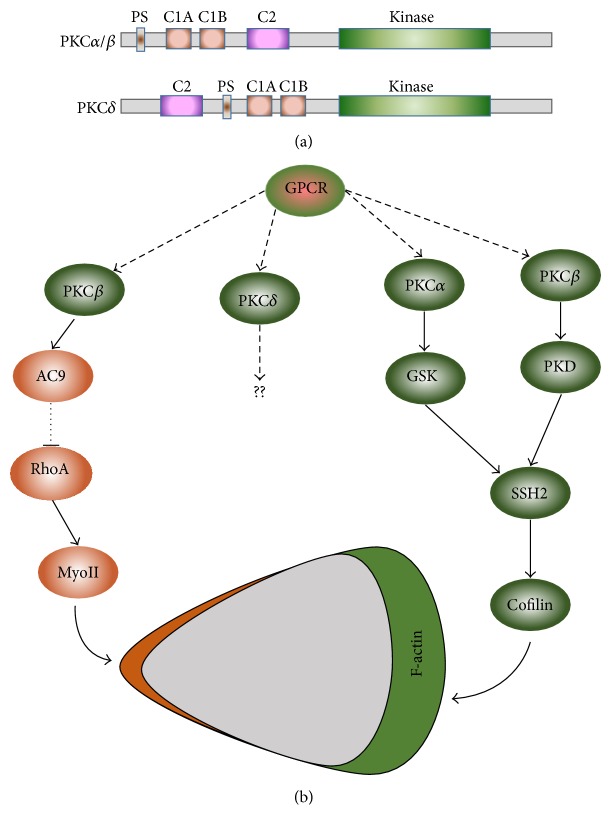
PKC*α* and PKC*β* play different roles in neutrophil polarization and chemotaxis. (a) Scheme shows the domain compositions of PKC*α*, PKC*β*, and PKC*δ*, which are expressed in neutrophils. All three PKC isoforms have an NH_2_-terminal regulatory domain that is joined through a flexible linker to a conserved COOH-terminal kinase domain that binds ATP and substrates. PKC regulatory domains contain a pseudosubstrate domain (PS) that maintains the enzyme in an inactive conformation. Membrane targeting modules (C1A-C1B-C2 domains) control the subcellular localization of the enzyme. (b) Scheme shows the signaling pathways in which PKC*α* and PKC*β* play essential roles in maintaining the polarization and chemotaxis of neutrophils.

PKC isoforms share a similar overall structure consisting of an NH_2_-terminal regulatory domain joined through a flexible linker to a conserved COOH-terminal catalytic domain that binds ATP and substrates [45]. The regulatory domain of PKC contains a pseudosubstrate domain that maintains the enzyme in an inactive conformation and membrane-targeting modules that control the subcellular localization of the enzyme. PKC isoforms are subclassified based on these membrane-targeting modules. Neutrophils express PKC*α*, PKC*β*I, PKC*β*II, and PKC*δ* [46]. PKC*α*, PKC*β*1, and PKC*β*II are conventional PKCs and contain tandem C1A/C1B motifs that bind diacylglycerol (DAG) or phorbol esters (such as PMA), a C2 domain that binds anionic phospholipids in a Ca^2+^-dependent manner, and a Ser/Thr kinase domain (Figure 2(a)). PKC*δ* is a novel PKC that contains a nonfunctional C2 domain and therefore is insensitive to Ca^2+^. Various stimuli activate all four PKC isoforms, and the activation of PKC is required for the oxidative burst of neutrophils [12, 47, 48]. PKC*α*, PKC*β*, and PKC*δ* phosphorylate all phosphorylation sites on p47phox [47, 49]. However, it is PKC*α* and PKC*δ*, but not PKC*β*, that play essential roles in fMLP- and PMA-induced superoxide generation in neutrophils or HL60 cells [48, 50], indicating that different PKC isoforms perform specific functions in neutrophils. The isoform-specific functions of PKCs have long been missing in the signaling pathways of neutrophil chemotaxis.

PKC*α* and PKC*β* share remarkable similarities in molecular composition, structure, and activation mechanism (Figure 2(b)). In resting neutrophils, both of them localize in the cytosol. Uniformly applied chemoattractant induces membrane translocation and subsequent activation of PKC*α* and PKC*β* in a PLC-dependent manner, indicating that the binding of DAG to their C1A domain serves as the major determinant for membrane translocation and activation [21, 23]. However, PKC*α* and PKC*β* interact with and activate different effectors to regulate SSH2 activity. GSK3, a substrate of PKC*α*, is active in resting neutrophils and phosphorylates SSH2 to decrease its cofilin phosphatase activity, in turn, leaving cofilin in an inactive phosphorylated state [21]. Upon fMLP stimulation, PKC*α* phosphorylates GSK3 and inhibits its activity, consequently increasing SSH2 activity and the activity of its target cofilin. Recently, it has been shown that PKC*β* interacts with and activates PKD1, and PKD1 phosphorylates SSH2 and inhibits its cofilin phosphatase activity [23]. By interacting with different effectors, both PKC*α* and PKC*β* regulate cofilin activity in order to regulate actin-based protrusion in the leading edge of chemotaxing cells. It was also reported that an mTORC2-specific activation of PKC*β*II regulates myosin II activity in the trailing edge of cells [51]. The authors revealed mTORC2-specific phosphorylation sites of PKC*β*II on its C-terminus. Point mutation of these sites resulted in impaired membrane translocation of PKC*β*II upon fMLP stimulation, providing an alternative membrane-targeting mechanism in addition to PLC signaling. The authors identified adenylyl cyclase 9 (AC9) as a downstream effector of PKC*β*II activation. Adenylyl cyclases (ACs) are activated and produce cAMP upon chemoattractant stimulation in both* D. discoideum* and neutrophils [52, 53]. In chemotaxing cells, cAMP is spatially restricted to the back of the cells to specifically regulate trail retraction and contraction in a MyoII-dependent manner [51–54]. This finding might also provide an example of how neutrophils utilize one common upstream activation pathway to precisely coordinate actin-based protrusion in the leading front and myosin II-based contraction in the trailing edge.

PKC*δ* translocates to the plasma membrane through the binding of its C1a domain with DAG or phorbol esters [8] and is involved in the oxidative burst in neutrophils [7, 55]. Recently, it has been reported that PKC*δ* is required for neutrophil transmigration mediated by IL-1*β* and fMLP (integrin-dependent), but not IL-8 (integrin-independent), by regulating adherence of neutrophils [4]. However, the molecular mechanism of PKC*δ*'s function in neutrophil chemotaxis remains unclear. In corneal epithelial cells, PKC*δ* mediates CAP37 (neutrophil-derived granular protein) induced chemotaxis [56]. In fibroblast migration and pulmonary fibrosis development, mTORC2-mediated PKC*δ* phosphorylation and cell migration downstream of G*α*12 have been reported [14]. Neutrophils express G*α*12, which localizes and reinforces signaling networks at the trailing edge of cells [13]. It would be interesting to know whether similar signaling pathways exist in neutrophils.

## 4. **PKD1 Is an Effector of the PLC/PKC Signaling Axis in Neutrophil Chemotaxis**

Protein kinase D (PKD) belongs to a family of serine/threonine kinases that play critical roles in many physiological processes, including cell growth, protein trafficking, and lymphocyte biology [26]. All three PKD isoforms are highly expressed in neutrophils [46]. The essential role of PKDs in neutrophil chemotaxis has only recently been revealed [23].

**Figure 3:**
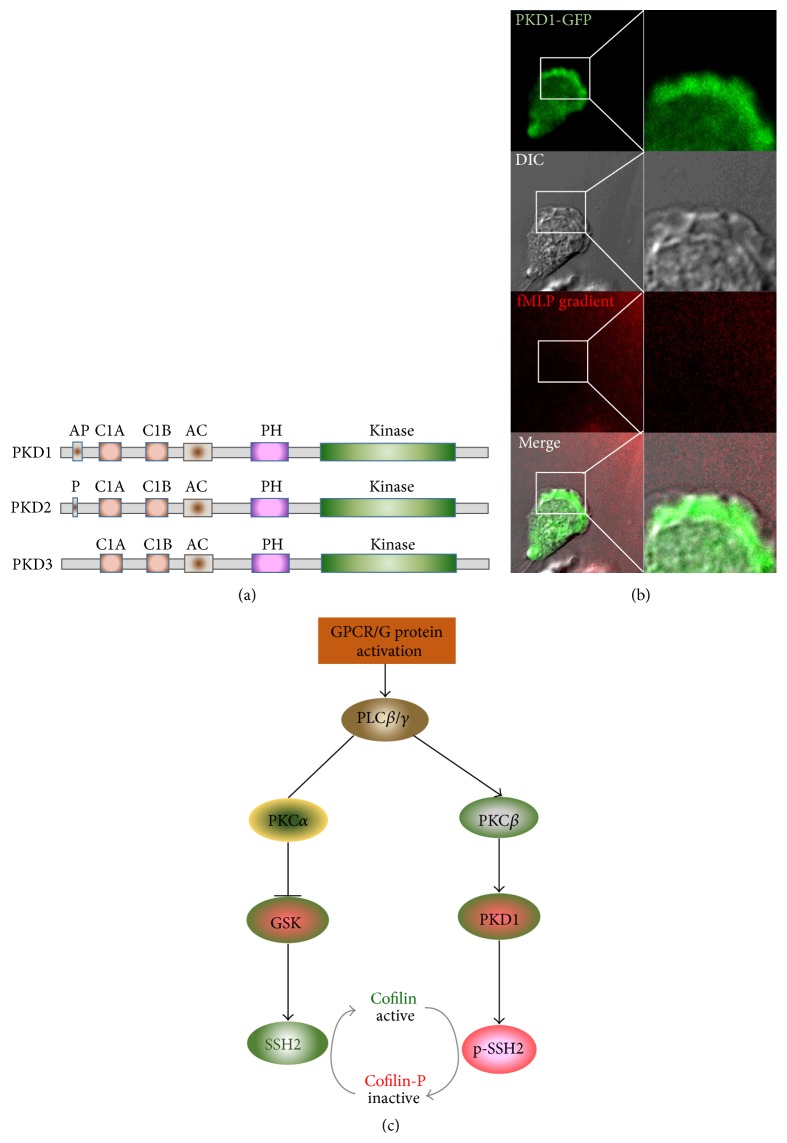
PKD, a direct effector of the PLC/PKC axis, is required for neutrophil chemotaxis. (a) Scheme shows the domain compositions of the three isoforms of the PKD family, PKD1–3. All three PKD isoforms have a conserved N-terminal C1A-C1B-AC-PH domain connected to a serine/threonine kinase domain at the C-terminal. The C1A domain binds to DAG for membrane targeting, while the C1B domain has a higher affinity for phorbol ester. C1A and C1B domains are separated by a long spacer, an acidic amino-acid-rich region (AC domain). The PH domain seals the kinase domain of PKD1 and inhibits its kinase activity. PKD1 also contains an alanine- and proline-rich region (AP domain) in its N-terminus while PKD2 has a proline-rich region (P domain) in its N-terminus. (b) PKD localizes at the backside leading edge of chemotaxis cells. HL60 cells expressing PKD1-GFP (Green) chemotax in 100 nM fMLP gradient (Red). In order to visualize the gradient, 100 nM fMLP was mixed with fluorescent dye Alexa 594. A differential interference contrast (DIC) image is also shown in order to portray the protrusion area of the leading edge. (c) Scheme shows the signaling pathways in which PKD1 phosphorylates cofilin phosphatase SSH2, ultimately regulating cofilin activity in GPCR-mediated neutrophil chemotaxis.

PKD isoforms share a conserved structural motif, N-C1A-C1B-PH-KD-C, and display a high sequence homology, particularly in the catalytic domain and C1A and C1B domains (Figure 3(a)). The C1A domain binds to DAG for membrane targeting, while the C1B domain has a higher affinity for phorbol ester [26]. This explains the fact that chemoattractant stimuli trigger very similar dynamics of membrane translocation and cellular localization for all three PKDs in neutrophils [23]. However, the differences in the N-terminal region and in the regions flanked by the C1 and PH domains may confer isoform-specific functions [26]. PKD1 contains an alanine-proline-rich region (AP domain) at the N-terminus, while PKD2 has a proline-rich region (P domain). Interestingly, there was a distinct expression profile of PKD isoforms in a panel of leukocyte cell lines [23]. The expression pattern of these three isoforms is not affected by the knockdown of the other isoforms, excluding the possibility of functional compensation among the three isoforms. Accumulating evidence demonstrates the involvement of PKDs in a variety of cellular processes that contribute to cancer development [57]. It has also been shown that specific PKD isoforms are misregulated in several cancer types, including leukemia [57]. It is important to understand PKD isoform-specific functions in neutrophils.

PKD is activated by several mechanisms [57]. In one mechanism, PKD is activated by direct phosphorylation of two conserved serine residues in its activation loop by DAG-binding PKC isoforms. Subsequent autophosphorylation allows its full and sustained activation. In neutrophils, chemoattractant stimulation induces robust phosphorylation of PKD1 at Ser744/Ser748 (activation loop) [23]. Phosphorylation at both sites is severely inhibited by PKC inhibitor GÖ6983, indicating that activation of PKD is directly through phosphorylation at the activation loop by PKC. The author identified that PKC*β*II, a DAG-binding PKC isoform, interacts with PKD1 and is essential for neutrophil chemotaxis [23]. Phosphorylation at Ser916, an autophosphorylation site of PKD1, is also detected in response to chemoattractant stimuli, indicating that autophosphorylation of PKD1 also occurs in neutrophils following chemoattractant stimulation.

Membrane translocation of PKD is required for its activation in neutrophils. Membrane translocation of PKD1 is mediated through several mechanisms in response to various kinds of stimuli, such as growth factor, phorbol esters, and GPCR agonists [26]. In resting, neutrophil-like HL60 cells, all three PKD isoforms localize in the cytoplasm and nucleus. In contrast to its behavior in other mammalian cell lines, the kinase-inactive mutant PKD1 (K612W) also localizes in the cytoplasm of HL60 cells, indicating that kinase activity is dispensable for PKD's cellular localization. Uniformly applied chemoattractant stimulation triggers a robust membrane translocation of all three PKDs. In a chemoattractant gradient, PKD localizes at the rear of the leading edge of chemotaxing cells (Figure 3(b)). The kinase-inactive mutant of PKD is recruited to the leading edge of chemotaxing cells, indicating that kinase activity is not required for membrane targeting of PKD. Instead, membrane targeting is completely abolished by either treatment with PLC inhibitors or a mutation of the C1A domain (DAG-binding domain), which results in a decreased affinity toward DAG. This result indicates that the binding of C1A domain and DAG is the major determinant for membrane targeting of PKDs in neutrophils. DAG also recruits PKC*β* to the membrane, where PKC*β* phosphorylates and consequently activates PKDs [23]. Thus, translocation to the plasma membrane allows PKD1 to interact with its upstream activator, such as PKC*β*, to be phosphorylated and subsequently activated. After being activated, this membrane localization might also provide close proximity for interaction with its substrates.

## 5. **Downstream Effectors of the PLC/PKC/PKD Signaling Axis Regulate Neutrophil Chemotaxis**

Each isoform of PLC, PKC, and PKD might have its own interacting partners in a separated signaling pathway in diverse functions of neutrophils. In the following two paragraphs, we are going to focus on the downstream effectors of the PLC/PKC/PKD axis that are involved in the remodeling of F-actin-based cytoskeleton and the regulation of other crucial signaling components.

The family of actin-depolymerizing factor (ADF)/cofilin proteins is comprised of cofilin-1 (a nonmuscle type of cofilin), cofilin-2 (a muscle type of cofilin), and ADF (also known as destrin) in mammals [58]. Active cofilin severs actin filaments and creates new barbed ends for actin polymerization [59]. Cofilin also contributes to F-actin assembly by increasing the actin monomer concentration for polymerization and consequently increasing the turnover rate of actin filaments in cells [60]. Cofilin might also increase new barbed ends by its intrinsic nucleation activity [61]. However, phosphorylation is the best-studied mechanism of regulating cofilin activity. LIM kinases (LIMKs) and testicular protein kinases (TESKs) phosphorylate cofilin to deactivate it while slingshot proteins (SSHs) and chronophin dephosphorylate p-cofilin to activate it [58]. In neutrophils, chemoattractants mediate the rapid dephosphorylation of cofilin [62]. A chemoattractant-mediated PLC*β*/PI3K*γ*/GSK3 signaling pathway has been found to increase the activity of SSH2, which dephosphorylates and activates cofilin (Figure 3(c)) [21]. The activation cycle of cofilin is especially important at the leading front, where rapid polymerization and depolymerization of F-actin cytoskeleton are required. Hirayama and his coworkers used HL60 cells to study the cofilin activation cycle and demonstrated a clear activation cycle [20]. A recent study has identified SSH2 as the direct target of the PLC/PKC*β*/PKD signaling axis to regulate cofilin activity [23]. Taken together, GPCR activation triggers two pathways to control the cycle of cofilin activity. The cofilin activation cycle is essential for a rapid and coordinated cycling of F-actin polymerization and depolymerization at the leading edge of chemotaxing cells (Figure 3(c)). However, the signaling pathways and kinases that phosphorylate cofilin are still not fully understood in neutrophils. Future work is necessary to fully understand the regulation of cofilin activity upon chemoattractant stimulation. In the future, it will be particularly important to understand how spatiotemporally distinct signaling pathways control the rapid and precisely coordinated regulation of cofilin activity in the leading front of chemotaxing neutrophils. A live probe to visualize cofilin activity in migrating cells is urgently needed.

The PLC/PKC/PKD1 signaling pathway might also regulate the localization and functions of other key components involved in neutrophil chemotaxis. PI3Ks phosphorylate phosphatidylinositol(4,5)-biphosphate (PtdIns(4,5)P_2_ or PIP_2_) into phosphatidylinositol(3,4,5)-triphosphate (PtdIns(3,4,5)P_3_ or PIP_3_) and the phosphatase and tensin homolog (PTEN) converts PIP_3_ back to PIP_2_. Leading-edge localization of PI3K and trailing-edge localization of PTEN are key features of gradient sensing and polarization and are essential requirements for chemotaxis in neutrophils and* Dictyostelium discoideum* [15, 63–65]. It has recently been shown that PKD1 directly phosphorylates the p85*α* subunit of PI3K to enhance its interaction with PTEN, leading to polarized PTEN activity and thereby regulating neutrophil migration [66]. Moreover, PKD1 might also play a role in PTEN membrane localization. Membrane localization of PTEN is required for its function in both* D. discoideum* and neutrophils. The C2 domain of PTEN is required but not sufficient to recruit* D. discoideum* PTEN to the plasma membrane [67]. Li et al. have shown that small GTPase RhoA/Rock mediates PTEN membrane targeting in murine neutrophils [15]. Recently, Nguyen et al. generated a library that contains green fluorescent protein (GFP) fused to randomly mutated human PTEN and expressed the library in* D. discoideum* cells [68]. One cluster of mutations with an enhanced membrane association is located in the C-terminal tail phosphorylation sites. These results indicate that phosphorylation plays essential roles in PTEN membrane targeting [15, 68]. It is not clear whether PKD1 is responsible for the phosphorylation of these sites in PTEN. However, trailing-edge localization of PKDs has been reported [23]. It is of great importance to understand whether PKD is the kinase responsible for the phosphorylation of these sites in PTEN, because both PTEN and PKD have substantial functions in various types of cancer.

## 6. Concluding Remarks

In this review, we strove to summarize recent findings regarding novel functions of the classic PLC/PKC/PKD signaling axis in neutrophil chemotaxis. Future research should focus on revealing isoform-specific functions of PLC*β*, PLC*γ*, and PLC*ε* in GPCR-mediated neutrophil chemotaxis, specifically PLC isoform-specific activation and the function of downstream effectors such as PKCs.
